# Music genre classification with modified residual learning and dual neural network

**DOI:** 10.1371/journal.pone.0333808

**Published:** 2025-10-14

**Authors:** Mohsin Ashraf, Fazeel Abid, Muhammad Owais Raza, Jawad Rasheed, Shtwai Alsubai, Tunc Asuroglu

**Affiliations:** 1 Department of Computer Science, University of Central Punjab, Lahore, Pakistan; 2 Department of Computer Science and Information Technology, University of Lahore, Lahore, Pakistan; 3 Department of Computer Engineering, Istanbul Sabahattin Zaim University, Istanbul, Turkey; 4 Department of Software Engineering, Istanbul Nisantasi University, Istanbul, Turkey; 5 Research Institute, Istanbul Medipol University, Istanbul, Turkey; 6 Applied Science Research Center, Applied Science Private University, Amman, Jordan; 7 Department of Computer Science, College of Computer Engineering and Sciences in Al-Kharj, Prince Sattam Bin Abdulaziz University, Al-Kharj, Saudi Arabia; 8 Faculty of Medicine and Health Technology, Tampere University, Tampere, Finland; 9 VTT Technical Research Centre of Finland, Tampere, Finland; VIT Bhopal University, INDIA

## Abstract

Music Genre is an abstract property of music that can identify shared traditions and conventions. In the recent past, music genre classification has shown a significant role in MIR that has attracted the research community to draw attention all around the world. The subjective aspect of the genre makes it challenging to define, as it relies on listeners’ interpretation. Deep Neural architectures can be used to address the efficiency and accuracy issues of traditional music systems. This paper proposes an approach to improve the music genre classification tasks with modified residual learning and hybrid convolutional neural networks. This architecture exploits the Mel-Spectrograms as input, which compute the signals as perceived by humans. We use identical layers of CNN with different pooling techniques to give rich hidden information for classification. We trained our model with Mel-Spectrograms generated from music files and obtained an accuracy of 87.80% and 68.50% for the GTZAN and FMA datasets, respectively. Our results show that the performance of the proposed model is also comparable with the other state-of-the-art models.

## 1 Introduction

Recently, music classification has gained momentum due to the unstoppable increase in music styles and dynamics. It is undoubtedly necessary to structure and organize the music for several applications in MIR, such as auto-tagging, music recommendations, and classification. A typical music classification system consists of three consecutive stages: Preprocessing of the input signals, Feature Extraction, and Selection of Specific Features and Classification. Preprocessing involves segmenting the input signals, which can then be used to extract specific features. Preprocessing aims to make the signals efficient and compatible with the training model. MFCC and spectral features with rhythmic contents have been used to improve the system performance by incorporating the wavelet decomposition as described in [1]. However, handcrafted features are difficult to design and require skills in related fields and resourceful engineering. Furthermore, handcrafted features are challenging to generalize, as various features are separately determined for different tasks and contexts in each case. Then input data instance is assigned a class label from the output class space in classification. Some significant applications of MIR include multimedia [2], audio surveillance [3], and music genre classification [4].

In recent years, spectrogram image features (SIF), stabilized auditory images (SAI), and linear prediction coefficients (LPC) have been utilized for audio analysis. A spectrogram visualizes the frequency spectrum of audio over time [5]. Spectrograms generated from music files are rare, so the lower region determines the noise intensity, and the higher region determines the robust components for classification tasks. Recent work [6] has combined refined composite multi-scale permutation entropy (RCMPE) with CNNs to overcome the limitations of traditional time-domain approaches. By incorporating multi-scale and time–frequency features, the method achieved high accuracy, including 100% recognition of ship noise and mechanical fault signals.

Deep learning architectures have achieved remarkable results in different fields by employing neural networks, including the MIR applications [7,8]. The work in [9] presented a survey on Deep Learning models that established a view for CNNs to be better than other models for image and video data analysis. CNNs are appropriate to detect objects in remote sensing images, as demonstrated in [10]. Similarly, as the spectrogram represents the frequency spectrum of the input signal over the time axis, deep learning techniques are capable enough to extract features from spectrograms for classification. However, music signals can be less frequent and a feeble zone, and cause several patterns in spectrograms. However, CNN has achieved significant performance in music processing and computer vision and is considered an appropriate method for classifying spectrograms. The first layer of CNN architecture learns features from the image. The second layer is a pooling layer that reduces the dimensions of feature maps generated from the convolutional layer. The performance of CNN is based upon three properties mentioned in [11]. These properties are (Local Receptive Fields, LRF), (Shared Weight, SW), and Spatial Sub-sampling. LRF refers to the neuron response affected by a specific part of the 2D image. SW is used in CNN to reduce the overall parameters [12]. Finally, Sub-sampling reduces the resolution of the output feature map, which addresses the issues of shifts and distortion in the final result. Many of the works in literature [13,14] have utilized different features and performed experiments on GTZAN [15] dataset. For music analysis, this dataset has 10 different genres, each with its own distinct patterns. The Free Music Archive (FMA) [16] dataset has recently gained attention for its comprehensive metadata that is essential for music analysis. Larger FMA datasets are available, and the small-FMA version is very comparable to GTZAN in terms of features.

This study introduces a novel dual-path convolutional architecture that integrates modified residual learning with hybrid pooling strategies to improve feature diversity and model generalization. Unlike prior works that focus solely on standard CNNs or singular pooling methods, our model uniquely fuses spatial and temporal features across multiple abstraction levels. The main contributions of this work include the following:

- A hybrid pooling framework that combines max and average pooling in parallel CNN paths to capture both dominant and subtle spectro-temporal cues.
- The integration of modified residual learning to preserve mid-level features and enable deeper architectures without degradation.
- Comprehensive evaluation on two benchmark datasets (GTZAN and FMA), demonstrating significant performance improvements and robustness against overfitting.

The rest of this article is organized as follows: Sect 2 explains the literature review, and Sect 3 elaborates on the proposed architecture. The dataset description and experiments are mentioned in Sect 4. Discussion about results is available in Sect 5, and the article is concluded in Sect 6.

## 2 Related work

Many domains, including NLP, music classification (MGC), and computer vision, have benefited greatly from using DNNs. Convolutional Neural Network (CNN) is found to be the most appropriate architecture for image classification in the literature. This architecture has played a significant role by employing the visual domain features such as Mel-spectrograms as mentioned in [17]. After doing this research, it was discovered that CNN may be utilized to extract relevant music feature patterns from Mel-spectrograms without the need for extensive information on handcrafted feature engineering. However, this work did not perform well in training. Further, to design the CNN model according to how the human mind extracts features, a model was discussed in [18]. According to this research, CNN can analyze the filter dimensions in both the time domain and the frequency domain. In their experiments, they made use of the Mel-Spectrum, SFM, and SCF characteristics, among others. According to [19], on the other hand, a model that performed computations on the modules was used to explore the representations with invariants and discriminative audio. Only the STFT with log representation was used to achieve robust results for music genre classification, employing stacked layers. The work in [20] investigated a CNN-based Network by utilizing the GTZAN dataset with small parameters using the global pooling technique. However, due to a lack of ability to localize the temporal features, they obtained a test accuracy of only 70.60%.

In [21], it was proposed to use a combination of convolutional neural networks and recurrent neural networks for the classification of music genres. The studies were conducted on the Million Song Dataset (MSD), which yielded moderate accuracy; however, due to the sequential nature of the RNN, model training took a considerable amount of time to complete. They also made use of the song length, which was approximately 29 seconds for each music piece. Using this segment length is not recommended for a number of different applications. Another work in [22] has presented a method for semantically modeling music samples that use CNNs with several max-pooling layers. Their suggested method could benefit from adding more layers to provide a strong representation of the music. Similarly, in [23], the authors proposed the CNN architecture incorporated with the residual network. Their experiments on the PMG-dataset exhibited an accuracy of 86% due to the lack of training samples. Further, a suggested model in [24] exploited a CNN-based architecture to classify the FMA dataset and found limited performance by achieving 66.4% accuracy. Furthermore, work in [25] made use of CNNs to extract learned features directly from music signals. They discovered that CNNs were more effective at detecting frequency patterns without the need for manual intervention. However, this approach lacked the visual domain expertise necessary to perform well with spectrograms, for example. [26] introduces a novel music genre classification method using functional data analysis (FDA) and adaptive Fourier decomposition (AFD), followed by support vector machine (SVM) classification. Experiments on GTZAN and FMA small datasets show it outperforms existing methods. [27] research introduces a method combining Capsule Neural Networks (CapsNet) with the upgraded Ideal Gas Molecular Movement (UIGMM) optimization algorithm to improve music genre classification. Evaluated on ISMIR2004, GTZAN, and Extended Ballroom datasets, showing its potential as a robust tool for music genre classification. [28] presents a zero-shot music tagging system using a joint music and language attention (JMLA) model, achieving 64.82% accuracy on the GTZAN dataset. The system outperforms previous methods and matches results on FMA and MagnaTagATune, leveraging a pre-trained audio encoder, Falcon7B decoder, and ChatGPT-enhanced dataset. [29] paper introduces a hybrid CNN-Transformer encoder (CNN-TE) model for music genre classification, outperforming existing CNNs on the GTZAN and FMA datasets with fewer parameters and faster inference. Using convolutional neural networks, the researchers in [17,30,31] were able to achieve substantial results, which prompted us to develop a novel method based on CNN layers that combines multiple pooling strategies in order to increase the accuracy of music classification. Researchers in [32] present an improved Vision Transformer model that combines convolutional networks and channel attention to better extract features from Mel spectrograms for music genre classification. The approach enhances precision and efficiency compared to earlier methods. The study [33] introduces a hybrid CNN-Transformer (CNN-TE) model for music genre classification using Mel spectrograms. The CNN captures low-level local features, while the Transformer encoder models global, high-level information. Tested on GTZAN and FMA datasets, the model outperforms many CNNs with fewer parameters and faster inference. [34] introduces the Broadcast Swin Transformer (BST), which enhances the Swin Transformer by better capturing low-level spectrogram features at multiple scales. Tested on the GTZAN dataset, BST outperforms state-of-the-art methods, showing strong potential for music genre classification.

Recent studies in diverse domains, including medical imaging and rainfall prediction, have successfully applied hybrid and optimization-driven deep learning techniques, demonstrating the versatility and cross-domain potential of such frameworks [35,36]. Although based on existing literature, CNNs and hybrid models have significantly improved the classification of music genres, current methods in the literature frequently only use one pooling technique, which restricts their capacity to capture both prominent and subtle spectro-temporal features. Performance deterioration also results from the inability of many deep architectures to maintain mid-level features due to insufficient residual learning. Furthermore, most studies rely on a single dataset for evaluation, lacking thorough testing to ensure generalization and robustness. To address these gaps, this work proposes a hybrid pooling framework that combines average and maximum pooling to capture a range of features. It also incorporates modified residual learning to preserve mid-level representations in deeper networks. It offers thorough testing on the GTZAN and FMA datasets, demonstrating improved accuracy and resistance to overfitting.

Table 1 summarizes key studies in music genre classification, grouped by approach. Early works focused on handcrafted or simple CNN-based features [17–25] extracted from Mel-spectrograms or STFTs, which often limited temporal and mid-level feature representation. Standard CNN models [20–24] improved hierarchical feature extraction using residual learning or max-pooling but achieved varying accuracy. Hybrid models combining CNNs with RNNs or Transformers [21,29,33,34] capture both local and global features, improving robustness and generalization. Capsule networks and optimization-based approaches [27] enhance feature extraction, while zero-shot or pre-trained attention models [28,32] leverage multi-modal encoders for further performance gains. Our proposed method builds on these insights by using hybrid pooling and modified residual learning to capture a diverse range of features and improve accuracy across GTZAN and FMA datasets.

**Table 1:** Comparison of music genre classification methods.

Approach	References	Dataset	Key Observations
Handcrafted/Feature-based	[17–19,25]	GTZAN, FMA	Extract features from Mel-spectrograms or STFT, limited temporal localization, moderate performance
CNN-based	[20,22–24]	GTZAN, PMG, FMA	Automatic feature extraction, residual learning improves mid-level feature preservation, accuracy varies 66-86%
Hybrid CNN-RNN/CNN-Transformer	[21,29,33,34]	GTZAN, FMA, ISMIR2004, Extended Ballroom	Combines local and global feature representation, better generalization, higher accuracy, slightly higher complexity
Capsule/Optimization-based	[27]	GTZAN, ISMIR2004, Extended Ballroom	Improved feature extraction and robustness using optimized networks
Zero-shot/Pre-trained Attention	[28,32]	GTZAN, FMA, MagnaTagATune	Leverages pre-trained encoders, multi-modal attention, moderate to high accuracy

## 3 Methodology

**Fig 1:**
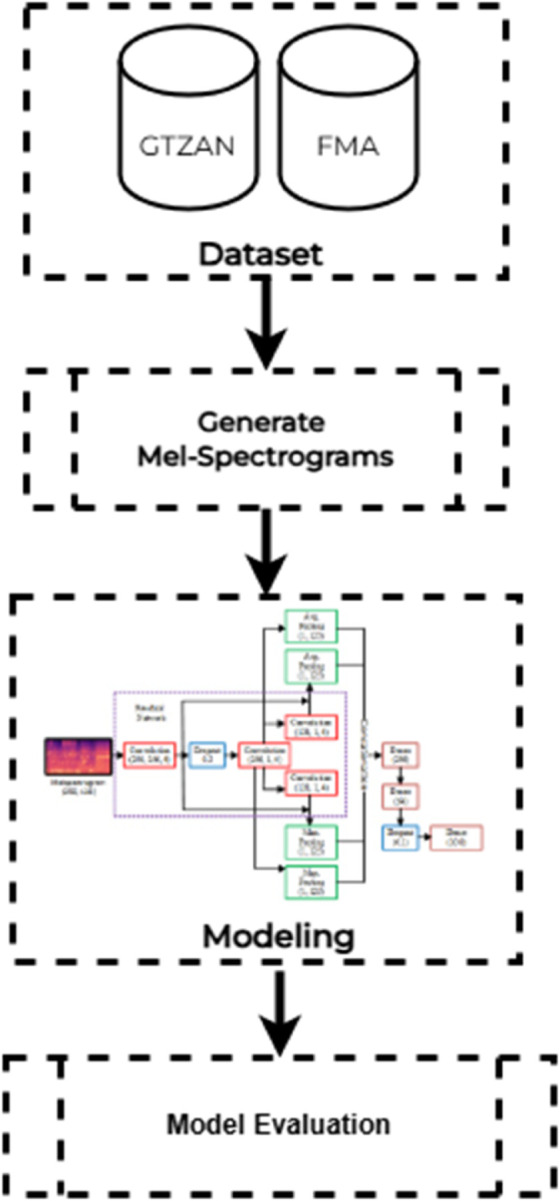
Proposed methodology for music genre classification using Dual CNN architecture with modified residual learning. It illustrates the overall methodology applied in the study, detailing the sequential process from dataset selection and preprocessing (Mel-Spectrogram creation) to deep learning model implementation (CNN with residual learning and hybrid pooling), and finally, the classification evaluation.

Fig 1 refers to the methodology applied in this study, which has four layers. Firstly, datasets are selected from these datasets, and dataset information is provided; the next step is to preprocess them and create Mel-Spectrograms. Deep learning modeling is applied once the data is in the form of Mel-Spectrograms. The approach used in this study is modified residual learning and hybrid CNNs, and the last models are evaluated using classification metrics.

### 3.1 Dataset

We evaluated our proposed model on two publicly available datasets: GTZAN [16] and FMA [17]. The GTZAN dataset contains 1000 music samples across 10 genres, with each song lasting 30 seconds. The FMA dataset is available in multiple versions (small, medium, full); we used the small FMA subset consisting of 8000 clips across eight genres, each 30 seconds long. All audio files are in MP3 format, sampled at 22,050 Hz, 16-bit depth, and mono channel. To increase sample diversity, we segmented all music files into 3-second clips and split them into training, validation, and test sets using an 8:1:1 ratio. Detailed per-genre distributions for both datasets are provided in Tables 2 and 3. To mitigate data imbalance and reduce overfitting, we employed five-fold cross-validation with random shuffling at each iteration.

**Table 2:** Instance distribution of the GTZAN dataset across training, validation, and test sets. The dataset contains a diverse set of musical categories. Thus, it shows the allocation of samples for each music genre in the GTZAN dataset. It ensures a balanced distribution for training, validation, and testing, supporting robust model evaluation.

Genre	Training Instances	Validation Instances	Test Instances
Blues	710	125	165
Classical	725	124	149
Country	734	125	138
Disco	710	128	160
Hip hop	715	126	157
Jazz	724	131	145
Metal	720	130	150
Raggae	715	132	153
Rock	710	120	170

**Table 3:** Instance distribution of the FMA dataset across training, validation, and test sets. The dataset contains a more diverse set of musical categories, presenting a greater challenge for classification. The allocation of samples for each music genre in the FMA dataset is shown to ensure a balanced distribution for training, validation, and testing, supporting robust model evaluation.

Genre	Training Instances	Validation Instances	Test Instances
Rock	7198	1254	1655
Pop	7235	1248	1653
Instrument	7208	1251	1420
International	7255	1305	1590
Hip-Hop	7150	1272	1575
Folk	7251	1378	1455
Experimental	7193	1400	1500
Electronic	7211	1395	1613

### 3.2 Creating mel-spectrograms

Neural Networks require an appropriate representation of the music signal for the feature extraction. For this purpose, we use Mel-Spectrograms of the music samples that illustrate the frequency variation along time, which are proficient in retrieving the unique characteristics of the input signal and decreasing the inconsistencies. Mel spectrograms are extracted using the short-time Fourier transform (STFT). STFT converts audio signals into time-frequency representations, capturing how frequencies change over time by applying the Fourier transform to overlapping windows of the signal. However, raw STFT spectrograms often emphasize linear frequency scales, which may not align with human auditory perception. To bridge this gap, we apply a Mel-scale transformation to the STFT output, resulting in Mel-spectrograms that provide a more perceptually relevant frequency distribution. This transformation compresses the frequency axis based on the Mel scale, emphasizing frequencies that are more distinguishable to the human ear and thus more meaningful for genre classification. These Mel-spectrograms serve as input to the neural network, offering a dense, informative representation of both spectral and temporal features. In our preprocessing pipeline, we used 30-second MP3 samples from the GTZAN and FMA datasets, resampled to a consistent sampling rate of 22,050 Hz. The STFT was computed with a window size of 2048 samples and a hop length of 512, followed by conversion to 128 Mel bands. Log scaling was applied to the Mel-spectrograms to compress dynamic ranges and reduce variance. This detailed preprocessing setup ensures the extraction of rich, discriminative features for robust classification. We compute the Mel-Spectrogram as the squared degree of STFT as in Eq (1):

$$STFT\;x\;\left(n\right)\left(m,\omega \right)=\sum_{n=-\infty }^{\infty }x[n]\omega \left[n-m\right]e^{-j\omega n}$$ (1)

Where ω[n] is a window function and *x*[*n*] refers to an input signal as in [37]. Typically, music is a sequence of data with a certain frequency at any given time instant, referring to the fact that it has a distinct pattern in it [38]. Further, these distinctive patterns of music signals are based on the frequency domain. On the other hand, the Mel-scale non-linear transformation represents the signals according to human perception, which is calculated in terms of power spectral density P (f,t). This transformation is also suitable for analyzing different time points (*t*_*i*_) and frequency (*f*_*j*_) during the variations. Mel-scale computes the frequency as:

$$Mel=2595*{log}_{10}\;(\;1+\;hertz/700\;)$$ (2)

The inverse can be calculated as:

$$Hertz=700*\left(10.0^{mel/2,595.0}-1\right)$$ (3)

The MP3-encoded 30-second files are used for both the GTZAN and FMA datasets as a result of this change. Training, validation, and test datasets are each partitioned into 8:1:1 subsets. We shuffle the samples to resolve the issue of reducing variance, making the model less overfit and remaining general [39]. When it comes to music signals, this study uses Mel-spectrograms to analyze their temporal structure, as mentioned in [40].

### 3.3 Modeling

**Fig 2:**
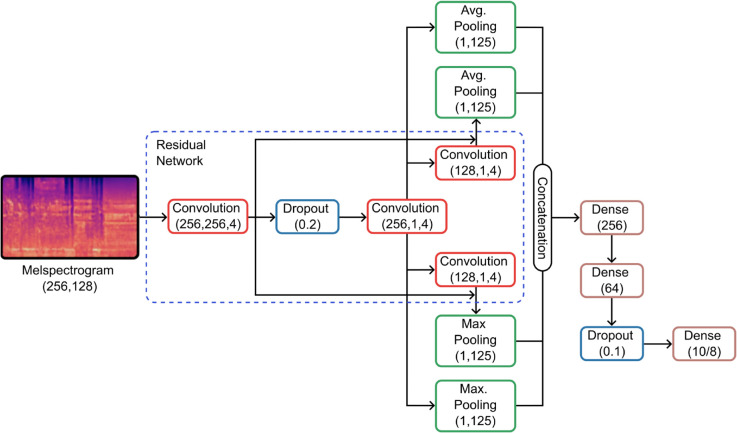
The detailed structure proposed Dual CNN architecture integrating different pooling techniques for music genre classification. The architecture incorporates dual convolutional layers to extract rich musical features from Mel-spectrograms, utilizing different pooling strategies to enhance feature representation, resulting in improved classification accuracy.

A number of applications have employed Convolutional Neural Networks to automatically extract features without the need for prior knowledge or human interaction. These networks have reduced the computations and dependency on the type of hand-crafted features needed for the classification tasks. However, the performance of the systems depends upon the architecture, including the number of layers, techniques, and hyperparameters. Therefore, system architecture must be carefully designed to achieve significant results. Fig 2 refers to the proposed Dual CNN Network Architecture with different pooling methods for the music genre classification task. This architecture has the following steps:

1. Dual Convolutional Neural Structure for Feature Extraction
2. Modified Residual Network
3. Concatenation and Model Output

#### 3.3.1 Dual convolutional neural structure for feature extraction.

In our work, the Mel-Spectrograms can be used to extract the important properties of a convolutional neural network that are most responsive to the final goal. The strides and padding are performed in the convolutional product to shrink the size and consider the edges of the images accordingly. Mathematically, an image is represented as:

$$dim(image)\;=\;(n_{H},n_{W},n_{C})$$ (4)

where *n*_*H*_ is height, *n*_*W*_ represents width, and *n*_*C*_ refers to the channels. The filter size (K) must have the same number of channels in the convolutional product.

$$dim(filter)\;=\;(f,\;f,n_{C})$$ (5)

where f is an odd dimension that permits every pixel to be centered in the filter among all other pixels around it. The following expression relates the filter and image in a convolutional operation:

$$Conv\left(I,K\right)=\sum_{i=1}^{n_{H}}\sum_{j=1}^{n_{W}}\sum_{k=1}^{n_{C}}K_{i.j.k}I_{x+j-1,k}$$ (6)

$$Conv\;\left(I,K\right)=\left(\left\lfloor \frac{n_{H}+2p-f}{s}+1\right\rfloor ,\;\left\lfloor \frac{n_{W}+2p-f}{s}+1\right\rfloor \right);s>0\;=(n_{H}+2p-f,n_{W}+2p-f);s=0$$ (7)

Pooling layers perform downsampling in each channel, keeping the number of channels (*n*_*C*_) intact and affecting only dimensions (*n*_*H*_, *n*_*W*_). No parameters are learned in pooling operations.

$$dim\;\left(pooling,(mel)\right)=\left(\left\lfloor \frac{n_{H}+2p-f}{s}+1\right\rfloor ,\;\left\lfloor \frac{n_{W}+2p-f}{s}+1\right\rfloor ,\;n_{c}\right);\;s>0\;=(n_{H}+2p-f,n_{W}+2p-f,n_{c});s=0$$ (8)

Further, batch normalization (BN) is performed with a sequence of operations:

$$\mu_{B}\leftarrow \;\frac{1}{m}\sum_{i=1}^{m}x_{i}$$ (9)

where *μ* is a mean, B (subscript) refers to the current batch, *m* is the number of inputs to the mini-batch, and *x*_*i*_ is the instance of input data. This operation results in a vector containing the mean of inputs within each batch.

$$\sigma_{B}^{2}\leftarrow \;\frac{1}{m}\sum_{i=1}^{m}{\left(x_{i}-\;\mu_{B}\right)}^{2}$$ (10)

In the second operation, variance $\sigma^{2}$ is computed from the standard deviation of the input.

$${\hat{x}}_{i}\;\leftarrow \;\frac{x_{i}-\;\mu_{B}}{\sqrt{\sigma_{B}^{2}+\;\in }}$$ (11)

The above operation shows normalization and zero-centering of the input. The numerical stability of the expression is assured with the smoothing term ∈ by avoiding division by zero. In the last operation, rescaling (*γ*) and shifting (*β*) of the input data are performed to obtain the final results of BN.

$$y_{i}\leftarrow {\gamma \hat{x}}_{i}+\;\beta \;\equiv \;{BN}_{\gamma ,\beta \;}(x_{i})$$ (12)

So, we perform the above convolutional operations and normalization techniques to extract the original characteristics from Mel-Spectrograms.

#### 3.3.2 Residual network block (ResNets).

The GTZAN dataset has been used to classify music genres using dual CNN layers, various pooling approaches, and residual learning. The Residual ResNets refer to an artificial neural network containing 2- or 3-layer skips with nonlinear functions like ReLU and BN. The key idea of ResNets is to understand the additive residual function F so that the layers are connected by the following equation.

$$x_{l}+1\;=\;x_{l}\;+F(x_{l},W_{l})$$ (13)

where *x*_*l*_ refers to the input feature to the *l*_*th*_ residual unit. *W*_*l*_ shows weights (biases) related to the *l*_*th*_ residual unit. In residual learning, suppose a neural network has to learn a *H*(*x*) function. So, instead of estimating *H*(*x*), ResNet explicitly permits the network to estimate the residual functions such as F(x):=H(x)−x (assuming the same dimensions for input and output). Further, outputs from the middle CNN layers are added to the output of the last CNN layer. It is possible to improve a DNN’s accuracy in image recognition tasks using residual learning. However, the addition of outputs can result in a loss of data. To solve this problem, we first feed CNN outputs to different pooling layers and concatenate them appropriately.

#### 3.3.3 Concatenation and output.

The concatenation of the outputs from ResNets has been discussed in [41], but the dual CNN layers with different pooling layers have not been used. Our proposed method can retain the important features of the input map even after pooling operations. This method enables CNN layers to extract distinct features, thereby optimizing the network for improved classification performance. Empirically, we have found that employing dual CNN layers with separate pooling layers after every convolutional layer helps us learn the features better without using a large number of parameters. All outputs from the pooling layers are concatenated and used as input to the next three dense layers with 256, 64, and 10 hidden units, respectively. These layers act as classifiers. ReLU [42] was utilized as an activation function in all of our layers except the final one. We utilized the SoftMax function in the last layer to allocate a label to a more appropriate class. The core function of SoftMax layers is to provide a range of probabilities for the sample classes by using specific hidden units. Mathematically, the probabilities are calculated as:

$$P\left(i_{j}|k,\theta \right)=\frac{exp\left(x_{j}\left(k,\theta \right)\right)\;}{\sum_{1\leq i\leq \left|X\right|}exp\left(x_{i}\left(k,\theta \right)\right)\;}1\leq j\leq \left|X\right|$$ (14)

where $x_{j}(k,\theta )$ is the input vector with *θ* showing parameters. *j* is the output instance of the class, and *X* is a class space. Exponentiation and summation of the input values are calculated. When this layer is finished, the output is a ratio between the input exponential and the sum of all of the exponential terms collected. We used regularizers and dropout layers to avoid overfitting in the neural networks. The first layer of our model employs *l*_2_ regularization with a penalty of 0.02. The other convolutional layers of the model use the same regularization with a 0.01 penalty. There are dropouts of 0.2 and 0.1 in the first convolutional layer and the last dense layer to prevent overfitting.

From the above model description, the following suggested algorithm explains the working. The proposed music classification system employs a Convolutional Neural Network (CNN) with residual connections to analyze Mel-Spectrogram representations of audio samples. Initially, a raw music sample is converted into a Mel-Spectrogram of shape (256,128), effectively capturing the signal’s time-frequency characteristics. The feature extraction process is performed through a sequence of convolutional layers. The first convolutional layer applies a large kernel of size (256,256,4) with a dropout of 0.2 to capture broad spectral patterns while reducing overfitting. Subsequent convolutional layers with kernel sizes (256,1,4) and (128,1,4) are incorporated into the residual network. One of these layers is combined with average pooling to enhance feature extraction. At the same time, another is integrated with max pooling, ensuring that both fine-grained and large-scale spectral features are retained. The outputs from all convolutional layers are concatenated and passed through a dense layer of size 256 for feature aggregation. A dropout layer (0.1) is applied to mitigate overfitting before the final classification step. The output layer utilizes a Softmax activation function to assign probabilities to predefined music categories. The combination of residual connections, various pooling strategies, and dropout regularization enhances the model’s ability to classify music genres accurately while maintaining robustness against overfitting.

**Algorithm 1 Music classification using residual CNN network.**

**Require:** Music sample *X*

**Ensure:** Predicted class label $\hat{y}$

1: **Preprocessing:** Convert the raw audio signal into a Mel-Spectrogram representation *S* of shape (256,128).

2: **Feature Extraction:** Pass *S* through a Residual CNN-based architecture as follows:

3:   (a) Apply 2D Convolutional Layer *C*_1_ with kernel size (256,256,4), followed by Dropout (0.2).

4:   (b) Apply another 2D Convolutional Layer *C*_2_ with kernel size (256,1,4).

5:   (c) Apply 2D Convolutional Layer *C*_3_ with kernel size (128,1,4), followed by Average Pooling.

6:   (d) Apply 2D Convolutional Layer *C*_4_ with kernel size (128,1,4), followed by Max Pooling.

7: **Feature Aggregation:** Concatenate outputs from all convolutional layers and feed into a Dense Layer of size 256.

8: **Regularization:** Apply Dropout (0.1) to the dense layer to prevent overfitting.

9: **Classification:** Use a Softmax activation function in the output layer to classify the music sample into one of the predefined categories.

10: **Return** Predicted class label $\hat{y}$.

### 3.4 Evaluation

#### 3.4.1 Accuracy.

Accuracy is the ratio of correctly predicted instances to the total number of instances:

$$\text{Accuracy}=\frac{\sum_{i=1}^{N}TP_{i}}{\sum_{i=1}^{N}(TP_{i}+FP_{i}+FN_{i})}$$ (15)

where *TP*_*i*_ represents the true positives for class *i*, *FP*_*i*_ represents the false positives, and *FN*_*i*_ represents the false negatives.

#### 3.4.2 Precision.

Precision measures the proportion of correctly predicted positive instances for each class:

$${\text{Precision}}_{i}=\frac{TP_{i}}{TP_{i}+FP_{i}}$$ (16)

The overall macro-averaged precision is:

$${\text{Precision}}_{macro}=\frac{1}{N}\sum_{i=1}^{N}{\text{Precision}}_{i}$$ (17)

#### 3.4.3 Recall.

Recall (Sensitivity) measures the proportion of actual positive instances that were correctly predicted:

$${\text{Recall}}_{i}=\frac{TP_{i}}{TP_{i}+FN_{i}}$$ (18)

The overall macro-averaged recall is:

$${\text{Recall}}_{macro}=\frac{1}{N}\sum_{i=1}^{N}{\text{Recall}}_{i}$$ (19)

#### 3.4.4 F1-Score.

The F1-Score is the harmonic mean of Precision and Recall:

$$F1_{i}=\frac{2\times {\text{Precision}}_{i}\times {\text{Recall}}_{i}}{{\text{Precision}}_{i}+{\text{Recall}}_{i}}$$ (20)

The overall macro-averaged F1-Score is:

$$F1_{macro}=\frac{1}{N}\sum_{i=1}^{N}F1_{i}$$ (21)

## 4 Experiments

This section consists of the experiments and results carried out to evaluate the proposed method described in the above section.

### 4.1 Model configuration and processing

Initially, we calculated the Mel-spectrogram of each input sample with the frame length of 1024, having a 50% overlap. Each Mel-spectrogram has 128 frames, and each frame has 256 frequency bins. The first convolutional layer of our network includes 256 kernels with a size of 256x4 without padding. The second convolutional layer also uses 256 kernels with a size of 1x4 but uses padding to equalize the input and output dimensions. The output of this second convolutional layer is fed into the third and fourth convolutional layers. Both of these layers use half the number of kernels (128) as used in the second convolutional layer (256). In these layers, padding is also used to avoid the reduction in the time axis. After performing convolution operations, all outputs are fed into pooling layers instead of adding them together. As the third and fourth convolutional layers are identical and have the same size and input, their outputs are given to pooling layers, such as max-pooling and average-pooling, to provide rich information about the distinct statistics available in Mel-spectrograms. This study employed both max-pooling and average-pooling in parallel on the outputs of identical convolutional layers. Max-pooling captures the most prominent features by focusing on the highest activations, which are often associated with strong spectral patterns in the Mel-spectrograms. In contrast, average-pooling captures the overall distribution of activations, which helps retain subtler yet informative patterns. By leveraging both types of pooling, our model benefits from a richer and more diverse feature representation. This design choice contributes to the observed performance improvements, as it allows the network to learn both localized and global statistical characteristics from the input features. Different pooling layers are used for the first and second convolutional layers. For the training of the proposed model, we recommend an Adadelta optimizer as suggested in [43] with the default learning rate of 1.0. We also used categorical cross-entropy as a loss function for our network. The size of each mini-batch was fixed to 50 samples. The model’s output is the genres’ probability for every music sample. The label of the song is associated with the music sample having the maximum probability. The performance measures to evaluate the model are classification accuracy, precision, recall, and F1-score. The obtained results are the mean of five iterations.

## 5 Results and discussion

**Fig 3:**
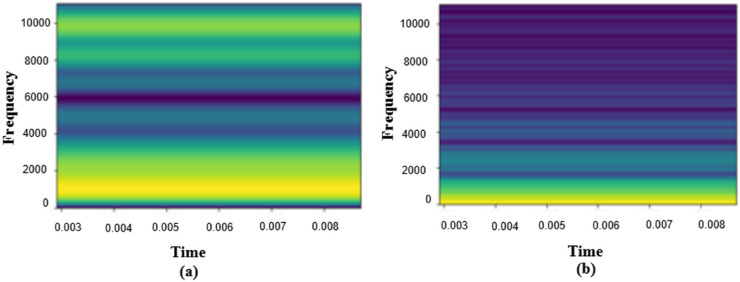
Comparison of Spectrogram representations: (a) Hertz-scale vs. (b) Mel-scale, demonstrating the energy distribution of Mel-scale for music classification. These two spectrogram representations are used for feature extraction in music classification. The Mel-scale transformation provides a more accurate representation of perceived sound frequencies, making it more suitable for genre classification tasks.

The suggested Dual CNN Network Architecture with two alternative pooling strategies (max-pooling and average-pooling) is analyzed in this section in detail. Fig 3 (a, b) represents a comparison between Spectrograms using Hertz-Scale and Mel-Scale. Mel-Scale has a better ability to show energy distribution along with frequency as it can undoubtedly show variations where most of the energy distillates. Music features are mostly related to the frequency domain instead of the time domain, so that Mel-Spectrograms can be more significant for music classification tasks. During investigations, we utilized multiple combinations of parameters, such as optimizer, learning rate, and batch size, and selected the best one from them.

**Fig 4:**
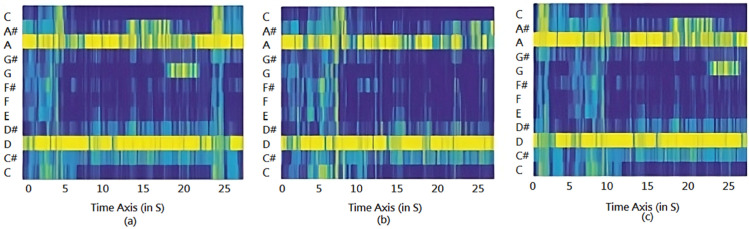
Feature representations extracted at different layers of the proposed CNN network, showcasing hierarchical learning from Mel-Spectrograms. Each layer captures different levels of musical information, from low-level frequency components to high-level genre-specific patterns.

Further, extracted features by convolutional layers of the proposed model, from top to bottom, can be seen in Fig 4(a, b, c). Our method, which utilizes different types of pooling layers, can extract distinct features with more statistical information to improve classification accuracy. Each pattern confirms to be unique and can be utilized for music classification tasks.

Table 4 presents the performance measures, such as precision and recall, for five iterations using GTZAN and FMA datasets with the proposed method. For the GTZAN dataset, iteration 5 yields the maximum values for precision and recall, specifically 93.10% and 89.70%, respectively. In contrast, iteration 1 shows the minimum values for precision and recall, such as 78.40% and 75.51%, respectively. Likewise, for the FMA dataset with the proposed method, iteration 2 yields the maximum scores for precision and recall of 76.10% and 68.66%, respectively, whereas iteration 4 yields the minimum values for precision and recall of 58.85% and 60.33%, respectively.

**Table 4:** Performance evaluation of the proposed Dual CNN model across five training iterations using GTZAN and FMA datasets. The study compares precision and recall scores across five training iterations to demonstrate the model’s learning improvements over multiple runs, highlighting variations in performance across different datasets.

Iterations	GTZAN Dataset		FMA Dataset	
	Precision	Recall	Precision	Recall
1	78.40	75.51	58.85	60.33
2	92.67	88.20	76.10	68.66
3	87.80	86.10	69.64	69.10
4	85.67	88.80	66.30	61.70
5	93.10	89.70	71.63	69.50

**Fig 5:**
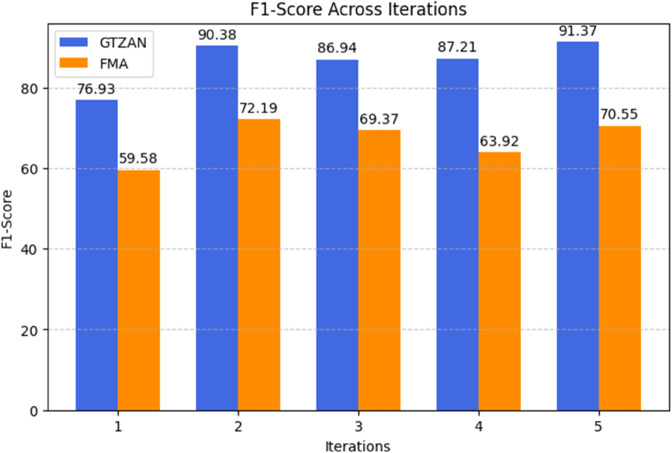
Performance comparison of five iterations for the GTZAN and FMA datasets, illustrating model stability and improvement trends. The graph shows the fluctuations in performance and the trend of model improvement, indicating how iterative training enhances classification accuracy.

The F1-score for the GTZAN and FMA datasets is compared over five iterations in the bar chart in Fig 5, and GTZAN consistently outperforms FMA. GTZAN shows a notable increase in Iteration 2 (90.38%) before stabilizing, starting at 76.93% and peaking at 91.37%. FMA starts lower at 59.58%, rises to 72.19% in Iteration 2, then varies, falling to 63.92% in Iteration 4 before marginally rising to 70.55% in Iteration 5. FMA struggles with more fluctuations, indicating its instability in maintaining F1-score improvements, whereas GTZAN maintains high performance with only slight variations.

The model’s performance in classifying each genre of music is highlighted in Table 5, which displays precision and recall values for various genres across the GTZAN and FMA datasets. Genres like classical, jazz, and metal exhibit perfect precision (1.00) and high recall for the GTZAN dataset, suggesting that these genres have very few false positives and very accurate predictions. Rock, hip-hop, and disco show strong but marginally lower recall and precision values, indicating some misclassification. Compared to other genres, blues and country have comparatively lower precision (0.89 and 0.68, respectively), suggesting a higher likelihood of misclassification. Performance varies, and fewer genres are assessed for the FMA dataset. While Instrumental (0.48, 0.55) exhibits the poorest performance, indicating difficulties in differentiating instrumental music, Folk (0.88, 0.83) and Rock (0.78, 0.73) show strong classification results. Pop and hip-hop in FMA perform noticeably worse than in GTZAN, suggesting that classification accuracy varies depending on the dataset.

**Table 5:** Genre-wise classification performance for the GTZAN and FMA datasets using the proposed Dual CNN model. The study calculated precision and recall values for each genre to identify well-classified genres, such as classical and jazz, while also indicating challenging categories, including instrumental and country.

Genre	GTZAN Dataset		FMA Dataset	
	Precision	Recall	Precision	Recall
Blues	0.89	0.75	-	-
Classical	1.00	0.95	-	-
Country	0.68	0.75	-	-
Disco	0.85	0.79	-	-
Hip Hop	0.79	0.82	0.68	0.63
Jazz	1.00	0.92	-	-
Metal	1.00	0.99	-	-
Pop	0.80	0.88	0.65	0.60
Reggae	0.92	0.89	-	-
Rock	0.85	0.83	0.78	0.73
Electronic	-	-	0.78	0.72
Experimental	-	-	0.61	0.65
Folk	-	-	0.88	0.83
Instrumental	-	-	0.48	0.55
International	-	-	0.62	0.67

**Fig 6:**
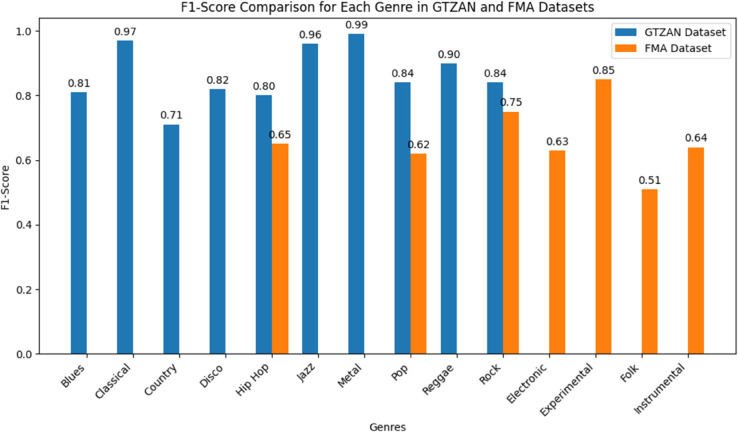
Genre-wise classification performance of the proposed Dual CNN Architecture on GTZAN and FMA datasets by providing insight into the model’s performance across diverse musical styles.

The bar graph in Fig 6 shows the F1 scores for the GTZAN and FMA datasets’ classification of musical genres. GTZAN (blue bars) displays F1 scores for ten genres, with Classical (0.97) and Jazz (0.96) exhibiting exceptional performances. The genres range from 0.71 (Country) to 0.99 (Metal). FMA (orange bars) shows scores for eight genres; Folk has the lowest score (0.51), while Experimental has the highest score (0.85). With the exception of Rock, where they tie at 0.75, GTZAN consistently outperforms FMA. Only Hip Hop, Pop, and Rock are present in both datasets.

Table 6 compares the performance of different methods in the GTZAN and FMA data sets for the classification of music genres, focusing on their accuracy with various feature extraction techniques. For the GTZAN dataset, the proposed method using Mel-Spectrograms achieved the highest accuracy of 87.80%, outperforming methods such as [18] 87.68% and [23] 86.00%. Similarly, on the FMA dataset, the proposed method ranked 68. 80% accuracy using Mel spectrograms, [44] 68. 07% and 66. 40% [24]. The results indicate that Mel-Spectrograms are effective for both datasets, with the proposed method consistently achieving superior performance compared to other feature extraction techniques such as Spectrograms [20] and MFCC [45]. Our work makes use of the advantages of employing only CNN with two pooling algorithms. We can finally conclude that the use of the Mel-Spectrograms and outputs taken from CNN layers with different pooling techniques incorporated with modified residual learning can significantly improve the accuracy of music genre classification. Although numerous studies have explored various feature extraction techniques and architectures, a fair comparison is challenging due to variations in dataset splits, pre-processing, feature extraction methods, and evaluation metrics. To ensure consistency, we only compare with studies using similar settings on GTZAN and FMA. This approach ensures methodological consistency, allowing for a more valid evaluation of the proposed method’s effectiveness under similar conditions.

**Table 6:** Comparison of classification accuracy between different deep learning models and feature extraction techniques on GTZAN and FMA datasets. It contrasts the accuracy of various models, demonstrating the superiority of the proposed CNN-based architecture with Mel-Spectrograms over traditional feature extraction methods such as MFCC and standard spectrograms.

Method	Features	Accuracy
**GTZAN Dataset**		
**Our Proposed**	**Mel-Spectrograms**	**87.80%**
Pons et al. [18]	Spectrograms	87.68%
N. Farajzadeh et al. [23]	Mel-Spectrograms	86.00%
P. Zhang et al. [22]	Mel-Spectrum, SFM, SCF	83.90%
C. Zhang et al. [46]	STFT with log representation	82.00%
A. Heakl et al. [20]	Spectrograms	82.00%
**FMA Dataset**		
**Our Proposed**	**Mel-Spectrograms**	**68.80%**
Guo et al. [44]	Echonest (Spotify)	68.07%
G. Sun et al. [24]	Mel-Spectrograms	66.40%
Chen et al. [47]	Mel-Spectrograms	65.23%
A. Heakl et al. [20]	Spectrograms	56.90%
Huang et al. [45]	MFCC, Mel-Spectrograms	44.30%

To assess the reliability of performance gains in this study, A paired t-test was conducted over five iterations. As shown in Table 7, both GTZAN and FMA datasets show statistically significant improvements. The GTZAN result is significant at the 90% confidence level (p = 0.075), while FMA shows stronger significance at the 90% level (p = 0.040). These findings confirm that the proposed method yields consistent and meaningful accuracy improvements across datasets.

**Table 7:** Statistical analysis of classification accuracy improvements using paired t-tests across five iterations. The table shows the mean accuracy improvement, 90% confidence intervals, and p-values for the GTZAN and FMA datasets. Improvements on both datasets are statistically significant at the 90% confidence level.

Dataset	Mean Improvement (%)	90% Confidence Interval (%)	p-value
GTZAN	3.71	[–0.61, 8.02]	0.075
FMA	5.99	[0.42, 11.56]	0.040

**Fig 7:**
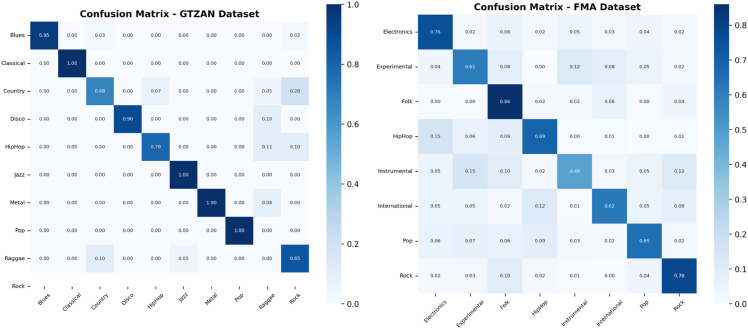
Confusion matrix for the proposed Dual CNN model using the GTZAN and FMA datasets, illustrating classification accuracy and common misclassifications. A strong classification accuracy can be noticed in certain genres like classical and jazz while indicating areas of confusion, such as misclassification between hip-hop and reggae.

Fig 7 shows the confusion matrix for the GTZAN and FMA datasets for our proposed model; the proposed model has an overall accuracy rate of 87.80%. It is accurate and performs well on three genres of music: classical, jazz, and metal. Although it misidentifies several genres, Hip-hop is projected to include a tiny percentage of reggae and rock in it. Similar to pop and disco samples, just a few rock samples were identified as such. When it came to predicting the country genre, this model performed the weakest. Only 68% of the samples were accurately predicted, with the remaining samples being jazz, pop, or rock genres. The work in [16] also confirms that country genre is difficult to classify and may require longer samples to capture for the right classification. For the FMA dataset, the overall accuracy is 68.50%. This model performs comparatively well in the folk genre with 86% accuracy. Electronic and rock genres have also been correctly predicted with an accuracy of 76% and 78%, respectively. It has shown the worst performance in the instrumental genre and has achieved only 48% accuracy. It can also be observed that a small percentage of every genre is wrongly predicted for most of the other genres. To improve the performance of the proposed model, more samples are required for the training with the optimized hyperparameter configurations.

### 5.1 Comparative performance analysis

The superior performance of the proposed Dual CNN model, particularly on the GTZAN dataset, can be attributed to three key design choices. First, the use of Mel-Spectrograms provides a perceptually meaningful representation of frequency components, improving the model’s ability to capture timbral features relevant to genre classification. Second, the dual pooling paths (max pooling and average pooling) complement each other by simultaneously capturing prominent local features and preserving overall statistical information, reducing information loss compared to single-path architectures. Third, the residual learning strategy facilitates improved gradient flow during training, leading to faster convergence and better generalization. However, the model exhibits relatively lower accuracy and greater fluctuation on the FMA dataset. This can be explained by the higher genre diversity, class imbalance, and intra-class variability present in FMA, which make feature learning more challenging. Similar difficulties in achieving stable performance on FMA have been reported by [24,44].

## 6 Conclusion

This article classifies the Music Genres with Modified Residual Learning using a Dual CNN Architecture incorporated with different pooling techniques. We utilize the Mel-Spectrogram of the music signals as input to scale the pitch magnitudes to extract features. Further, different pooling, i.e., Average and Max, get the feature vectors as input from the convolutional layers. Our proposed methodology demonstrates that applying the outputs of dual convolutional layers, in which the output of modified CNN layers is a total output based on residual learning, improves the Music Genre Classification. This study used GTZAN and FMA datasets for evaluation. These datasets were used to ensure fair and standardized evaluation because these datasets offer sufficient genre diversity and are commonly used in related work, enabling consistent comparisons. Future work will explore additional datasets to assess the generalizability of the proposed method further. While this work focuses on STFT-based Mel spectrograms for input representation, several alternative spectro-temporal analysis techniques have been proposed in the literature with demonstrated benefits for audio processing tasks. Wavelet-based signal processing and analysis techniques, including Empirical Mode Decomposition and Central Tendency Measures, have been extensively applied in recent studies [48,49]. Wavelet transforms provide multi-resolution time-frequency analysis, capturing both transient and stationary features, as in the case of voice classification [50] and parkinson disease detection from voice [51]. Single Frequency Filtering (SFF) decomposes a signal into instantaneous frequency trajectories at individual frequencies, offering fine-grained resolution and robustness in speech, speaker, and pathological voice analysis [52–55]. Zero-Time Windowing (ZTW) extracts high-resolution spectral envelopes and harmonic structures by applying analysis windows at zero time instants, which has proven effective for tasks such as emotion recognition, voiced/unvoiced detection, and pathological speech assessment [56–59]. Incorporating such representations in music genre classification could potentially enhance discriminative feature learning and improve generalization across diverse datasets. While these methods are beyond the current scope, their integration presents a promising avenue for future work.

The proposed framework has the potential for wider uses in audio content analysis, including speech emotion recognition, instrument detection, environmental sound classification, and music emotion recognition, in addition to music genre classification. These tasks can take advantage of the improved feature extraction capabilities shown in this work and also benefit from detailed time-frequency representations. The proposed architecture can also be applied to various domains of audio content processing. Most importantly, all areas of MIR research could benefit from a strenuous effort to develop wisely annotated music datasets that contain diverse metadata such as mood, lyrics, composer, and other related terms.
